# A Novel Hexose Transporter ChHxt6 Is Required for Hexose Uptake and Virulence in *Colletotrichum higginsianum*

**DOI:** 10.3390/ijms22115963

**Published:** 2021-05-31

**Authors:** Qinfeng Yuan, Yaqin Yan, Muhammad Aamir Sohail, Hao Liu, Junbin Huang, Tom Hsiang, Lu Zheng

**Affiliations:** 1 Hubei Key Laboratory of Plant Pathology, Huazhong Agricultural University, Wuhan 430070, China; yuanqf1989@163.com (Q.Y.); zkyyanyaqin@163.com (Y.Y.); amirsohail306@gmail.com (M.A.S.); hl210@mail.hzau.edu.cn (H.L.); junbinhuang@mail.hzau.edu.cn (J.H.); 2 School of Environmental Sciences, University of Guelph, Guelph, ON N1G 2W1, Canada; thsiang@uoguelph.ca

**Keywords:** *Colletotrichum higginsianum*, hexose transporter, hexose uptake, virulence

## Abstract

*Colletotrichum higginsianum* is an important hemibiotrophic plant pathogen that causes crucifer anthracnose worldwide. To date, some hexose transporters have been identified in fungi. However, the functions of hexose transporters in virulence are not clear in hemibiotrophic phytopathogens. In this study, we identified and characterized a new hexose transporter gene named *ChHxt6* from a T-DNA insertion pathogenicity-deficient mutant G256 in *C. higginsianum*. Expression profiling analysis revealed that six *ChHxt* genes, *ChHxt1* to *ChHxt6*, exhibited specific expression patterns in different infection phases of *C. higginsianum.* The *ChHxt1* to *ChHxt6* were separately deleted using the principle of homologous recombination. *ChHxt1* to *ChHxt6* deletion mutants grew normally on PDA plates, but only the virulence of *ChHxt4* and *ChHxt6* deletion mutants was reduced. *ChHxt4* was required for fungal infection in both biotrophic and necrotrophic stages, while *ChHxt6* was important for formation of necrotrophic hyphae during infection. In addition, ChHxts were functional in uptake of different hexoses, but only *ChHxt6*-expressing cells could grow on all five hexoses, indicating that the ChHxt6 was a central hexose transporter and crucial for hexose uptake. Site-directed mutation of T169S and P221L positions revealed that these two positions were necessary for hexose transport, whereas only the mutation Thr169 caused reduced virulence and defect in formation of necrotrophic hyphae. Taken together, *ChHxt6* might regulate fungal virulence by modulating the utilization of hexose.

## 1. Introduction

The hemibiotrophic ascomycete fungus *Colletotrichum higginsianum* is an important pathogen that causes anthracnose on various cruciferous plants and leads to serious economic losses worldwide [1]. In the infection cycle, the conidia of *C. higginsianum* attach onto the host surfaces, germinate and produce dark appressoria. Then, the fungus penetrates epidermal cells and generates large bulbous biotrophic hyphae in the first infected living cell. Finally, thin necrotrophic hyphae are produced which grow to kill host tissues, resulting in formation of necrotic leaf lesions [2].

In fungi, the system for uptake and utilization of carbon sources is highly conserved and enables fungal cells to sense and respond to extracellular conditions [3,4]. The hexose transporter (HXT) cascade is one of the ubiquitous signaling systems in *Saccharomyces cerevisiae* [5,6]. So far, a few plasma membrane-localized carbohydrate transporters have been identified in other fungi including the biotrophic fungi *Uromyces fabae* (the causal agent of broad bean rust) [7,8], *Ustilago maydis* (the causal agent of corn smut) [9,10], and *Puccinia striiformis* (the causal agent of wheat stripe rust) [11,12], the symbiotic glomeromycotan soil fungus *Geosiphon pyriformis* [13], as well as the hemibiotrophic maize pathogen *Colletotrichum graminicola* [14], and the rice blast fungus *Magnaporthe oryzae* [15]. The proteins identified in *U. fabae* (UfHXT1) and *G. pyriformis* (GpMST1) were monosaccharide transporters that restored the growth of hexose uptake-deficient yeast strains on mannose and glucose, and the lost transport activities in yeast [8,13]. The *U. maydis* transporter UmSRT1 is specific for sucrose and the *UmSRT1* deletion mutants of *U. maydis* showed strongly reduced virulence on maize plants [9]. Direct uptake of sucrose by the UmSRT1 protein of *U. maydis* was interpreted as a mechanism to avoid apoplastic signals (glucose) potentially recognized by the host [9]. The gene *Hxt1* identified in *U. maydis* encodes a high-affinity transporter for glucose, fructose, and mannose, and disruption of *Hxt1* resulted in reduced growth on these substrates and showed decreased symptom development on maize [10]. In *P. striiformis*, an invertase PsINV is unique in fungi and important for sugar uptake during infection. PsINV is abundantly secreted into the host to secure sugar absorption for *P. striiformis,* and silencing *PsINV* inhibits growth and development as well as sporulation [11]. Hexose transporter in *P. striiformis PsHXT1* is required for pathogenicity through sugar uptake. PsHXT1 is a glucose–proton symporter, silencing *PsHXT1* restricts normal growth and development of *P. striiformis* during infection [12]. In the hemibiotrophic phytopathogen, *C. graminicola*, hexose transporter genes *CgHXT1*-*CgHXT5* exhibit various expression profiles during infection of maize and in hexose uptake [14]. MoST1 in *M. oryzae* plays a specific role for conidiation and mycelial melanization [15]. However, the functions of hexose transporters in virulence are not clear in hemibiotrophic phytopathogens.

In this study, we identified and characterized a new HXT gene named *ChHxt6* from a T-DNA insertion pathogenicity-deficient mutant, G256, in *C. higginsianum*. Deletion of *ChHxt6* showed reduced fungal virulence and caused less necrotrophic hyphae formation. Furthermore, ChHxt6 was found to be important for uptake of multiple hexoses. Site-directed mutants of Thr169 and Pro221 positions on *ChHxt6* also revealed deficiencies in virulence and hexose transportation, suggesting that *ChHxt6* is essential for virulence possibly mediated by affecting hexose uptake in the switch from biotrophy to necrotrophy.

## 2. Results

### 2.1. Identification of a New Hexose Transporter Gene in C. higginsianum

**Figure 1:**
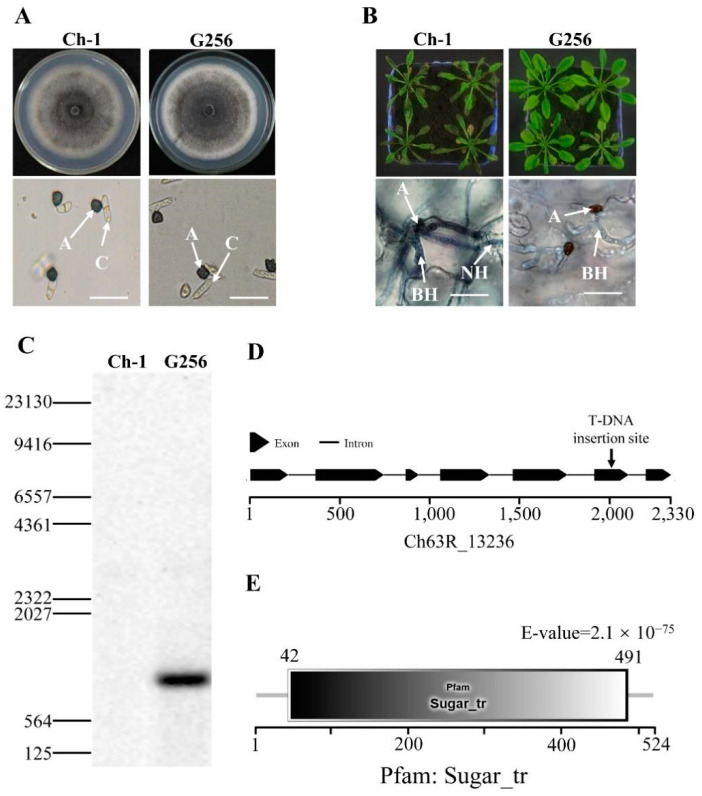
Identification of T-DNA insertional mutant G256 in *C. higginsianum*. (**A**) Colony morphology and conidial germination of G256. The top pictures show colony morphology of strains cultured for 7 days on PDA in darkness at 25 °C, and the bottom pictures show conidial germination on plastic coverslips at 24 h. A: appressoria; C: conidia; bar = 20 μm. (**B**) Virulence test of G256. The pictures on the top show the symptoms caused by spraying inoculation of conidia on 6-week Arabidopsis for 4 days; the pictures at the bottom show observation of infection structures under microscope at 48 h post inoculation, A: appressoria; BH: biotrophic hyphae; NH: necrotrophic hyphae. Bar = 20 μm. (**C**) Analysis of T-DNA copies inserted in G256 by Southern blotting. The DNA probe was designed for hph. (**D**) T-DNA was inserted in exon of hexose transporter coding gene Ch63R_13236. (**E**) Ch63R_13236 encoding protein contained a conserved Sugar_tr domain.

The mycelial growth of *C. higginsianum* T-DNA insertion mutant G256 was not noticeably different from the wild-type on PDA (Figure 1A). The conidial germination morphology of the mutant G256 also did not differ (Figure 1A). Interestingly, the virulence of G256 on *Arabidopsis* was reduced, and formation of necrotrophic hyphae was deficient (Figure 1B). Southern blotting with the hygromycin phosphotransferase (hph) gene probe indicated that only one T-DNA insertion copy was detected in the genome of the mutant G256 (Figure 1C). By using inverse PCR, the T-DNA insertion site was identified in the fifth exon of the CH63R_13236 gene (Figure 1D), which contained a Sugar_tr-conserved domain and was annotated as hexose transporter protein (called ChHxt6) (Figure 1E).

### 2.2. Characterizations of Six Hexose Transporter Genes in C. higginsianum

Since previous study revealed that five CgHXT proteins in *C. graminicola* might have conserved functions in plant pathogenic fungi during fungal pathogenesis (Lingner et al., 2011), we selected homologues of these five Hxt proteins (ChHxt1, ChHxt2, ChHxt3, ChHxt4 and ChHxt5) in *C. higginsianum* as well as ChHxt6 identified in this study for further function analysis.

**Figure 2:**
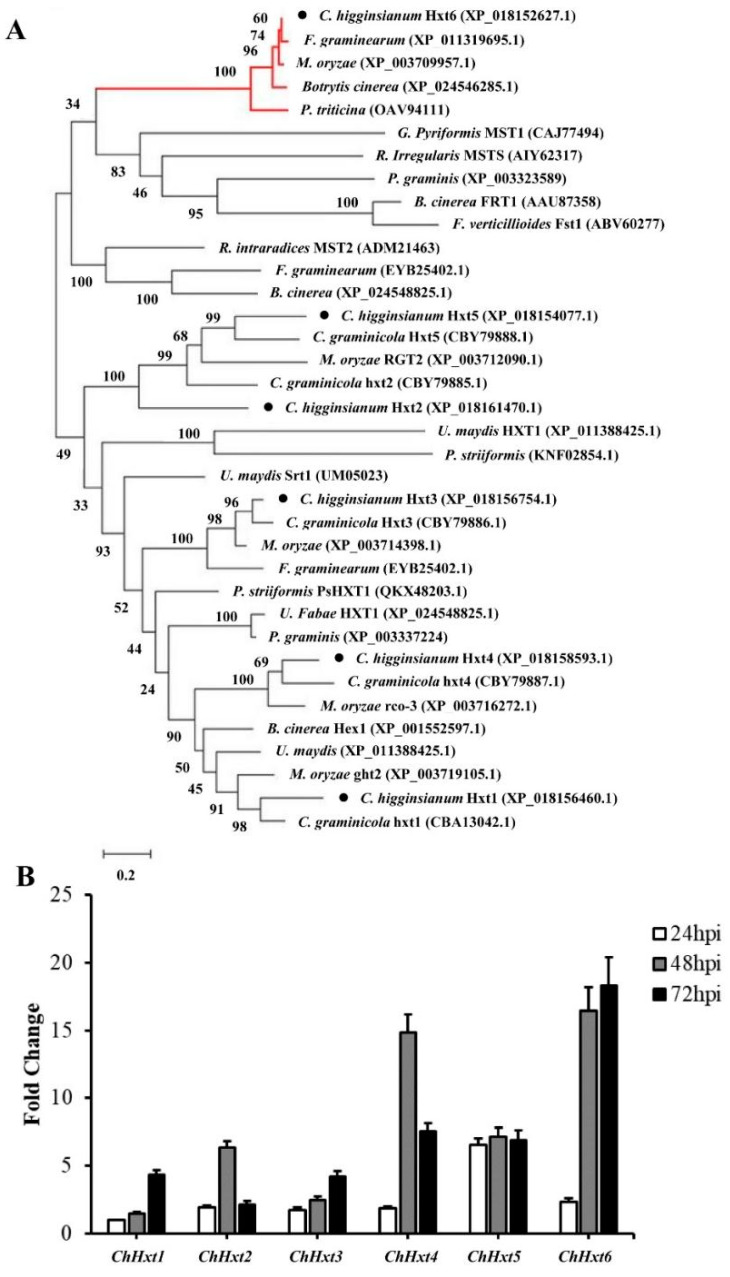
Characterization and expression profiles of six hexose transporter (HXT) genes in *C. higginsianum*. (**A**) Phylogenetic tree of six ChHxts and other fungal hexose transporters constructed using the Neighbor-Joining method. The accessions are *C. graminicola* (CBA13042.1, CBY79885.1, CBY79886.1, CBY79887.1, CBY79888.1, XP_008089954.1), *C. higginsianum* (XP_018156460.1, XP_018161470.1, XP_018156754.1, XP_018158593.1, XP_018154077.1, XP_018152627.1), *Botrytis cinerea* (XP_001551724.1, XP_001551177.1, XP_001558083.1, XP_024548825.1, XP_001552597.1, XP_024546285.1), *Fusarium graminearum* (XP_011318591.1, XP_011323525.1, EYB25402.1, XP_011328407.1, PCD34454.1, XP_011319695.1), *Puccinia striiformis* (KNF02854.1, KNE94049.1), *Magnaporthe oryzae* (XP_003712090.1, XP_003719105.1, XP_003714398.1, XP_003716272.1, XP_003709957.1), and *Ustilago maydis* (XP_011391314.1, XP_011388425.1, XP_011391901.1). The red lines at the top indicate the outgroup formed by ChHxt6 homolog. (**B**) Expression profiles of *ChHxt* genes at different infection stages on *Arabidopsis*. Conidia of *C. higginsianum* wild-type Ch-1 were sprayed onto *Arabidopsis* leaves, and leaves were collected at 24, 48 and 72 h post-inoculation for total RNA extraction and RT-qPCR analyses. Bars show means and standard deviations of the expression fold change (*n* = 3).

Phylogenetic analysis was performed with putative Hxts of functionally characterized proteins from some phytopathogenic fungi and functionally characterized HXT-type transporters (Figure 2A). The results demonstrated that the Hxt proteins form separate clusters together with HXT-type proteins from other phytopathogenic fungi. Closely related homologs for ChHxt1, ChHxt2, ChHxt3, ChHxt4 and ChHxt5 were found in *C. graminicola*. In addition, the *ChHxt6* gene identified from G256 T-DNA mutant was separated as outgroup, indicating that the ChHxt6 is a novel hexose transporter in *C. higginsianum*.

Expression of the six *ChHxt* genes during infection stages were analyzed by RT-qPCR (Figure 2B). *ChHxt5* was expressed stably at all infection stages. Expression levels of *ChHxt1* and *ChHxt3* at 72 hpi during necrotrophic stages were higher than other infection stages. *ChHxt2* and *ChHxt4* were highly expressed during the switch from biotrophy to necrotrophy (48 hpi). *ChHxt6* expression levels were significantly up-regulated at both 48 and 72 hpi. Expression profiling analysis revealed that *ChHxt1* to *ChHxt6* exhibited specific expression profiles in different infection phases of *C. higginsianum*, suggesting differences in functional characteristics for these six ChHxt proteins.

### 2.3. Deletion of the Six Hexose Transporter Genes in C. higginsianum

**Figure 3:**
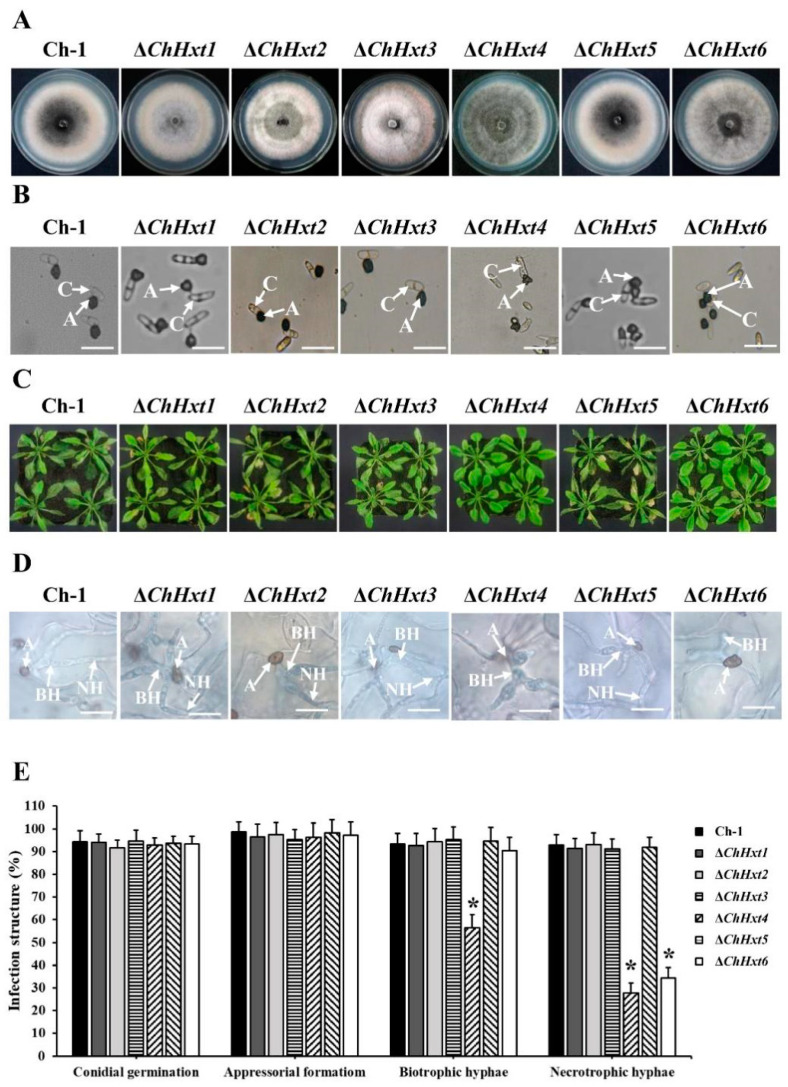
Characterization of *ChHxt1~ChHxt6* knockout mutants in *C. higginsianum*. (**A**) Colony morphology of the mutants. Wild-type and mutant strains were cultured for 7 days on PDA in darkness at 25 °C. (**B**) Conidial germination of the mutants. Conidia of the wild-type and mutants were cultured on plastic coverslips at 25 °C for 24 h, and conidia germinated to produce appressoria were observed under microscope. A: appressoria; C: conidia; bar = 20 μm. (**C**) Virulence test of the mutants. Observation of the symptoms on 6-week-old *Arabidopsis* leaves at 4 days post-inoculation. (**D**) Infection structures at 48 h post-inoculation. The *Arabidopsis* leaves inoculated conidia of the wild-type and mutants were collected at 48 h post inoculation, after decoloring with methanol: chloroform: glacial acetic acid (6:3:1, *v*/*v*/*v*) and staining with trypan blue (0.4%). Infection structures were observed under microscope at 48 h post-inoculation. A: appressoria; BH: biotrophic hyphae; NH: necrotrophic hyphae. Bar = 20 μm. (**E**) Generation rate of infection structures. To calculate the infection structure generation rate, at least 100 infection structures were observed, and each test was repeated three times. Bars indicate means and standard deviations of generation rates of infection structures. Asterisks represent significant differences between the Ch-1 and other strains by LSD test at *p* = 0.05.

The six Hxt genes, *ChHxt1* to *ChHxt6*, were separately knocked out using homologous recombination (Figure S1A). All deletion mutants were confirmed by PCR amplification (Figure S1B). Vegetative growth of all deletion mutants was similar to the wild-type (Figure 3A). Colonies of *ChHxt1*-*ChHxt3* deletion mutants were less melanized and had more aerial hyphae than the wild-type, whereas colonies of the *ChHxt4* deletion mutant were darker than the wild-type (Figure 3A). We further tested the appressorial formation of the mutants on artificial hydrophobic surfaces using polystyrene plastic coverslips. The wild-type and the *ChHxt* deletion mutants formed normal appressoria on plastic coverslips (Figure 3B).

### 2.4. ChHxt4 and ChHxt6 Are Important for Virulence in C. higginsianum

To test the pathogenicity of the deletion mutants, conidial suspension of each mutant was sprayed onto *Arabidopsis* plants, and the results showed that virulence of Δ*ChHxt4* and Δ*ChHxt6* were significantly decreased and formed fewer necrotic lesions on leaves at 4 days post inoculation (dpi) (Figure 3C). In contrast, *ChHxt1*, *ChHxt2*, *ChHxt3* and *ChHxt5* deletion mutants caused typical necrotic lesions as the wild-type.

Microscopic analysis demonstrated that most appressoria of the wild-type, Δ*ChHxt1*, Δ*ChHxt2*, Δ*ChHxt3* and Δ*ChHxt5* penetrated to form both bulbous biotrophic hyphae and thin filamentous necrotrophic hyphae (Figure 3D,E). In contrast, formations of biotrophic hyphae and necrotrophic hyphae of Δ*ChHxt4* were both significantly reduced, while less necrotrophic hyphae were found in Δ*ChHxt6* (Figure 3D,E). These indicated that *ChHxt4* was required for fungal infection in both biotrophic and necrotrophic stages, and *ChHxt6* was important for the formation of necrotrophic hyphae.

### 2.5. ChHxt6 Is a Central Monosaccharide Transporter for Hexose Uptake

**Figure 4:**
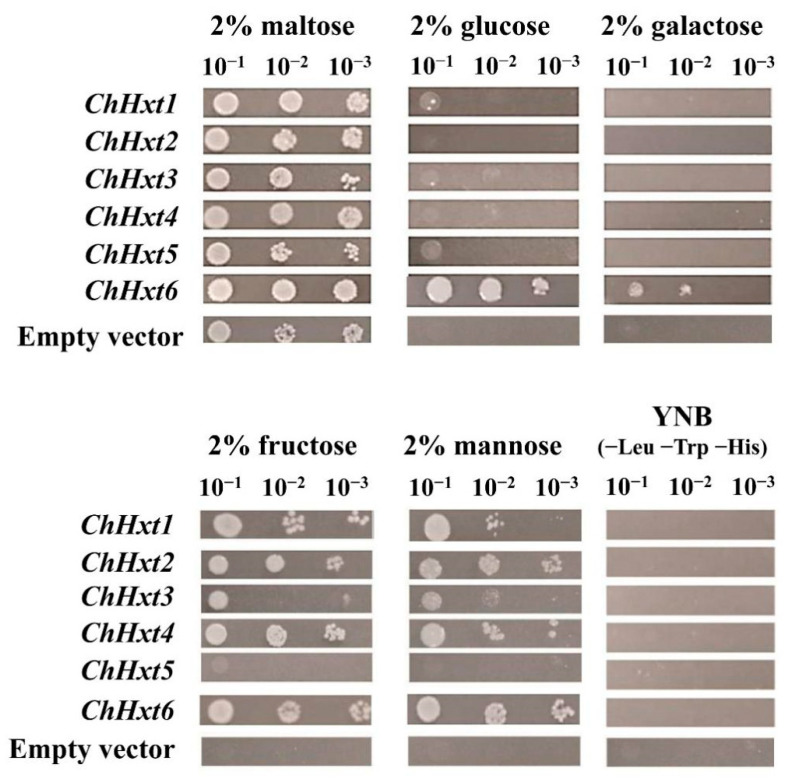
Growth of the *Saccharomyces cerevisiae* strain EBY.VW4000 (lacking all 20 identified hexose transporters) and modified EBY.VW4000 strains expressing different ChHxt genes (ChHxt1 to ChHxt6). Expression of ChHxt genes complemented the growth defect of EBY.VW4000 on YNB media supplied with different monosaccharides. Growth of yeast was observed and recorded after incubation at 28 °C for three days.

To further explore functional characterization of the hexose uptake of the six *ChHxt* genes, all six *ChHxt* genes were expressed in the monosaccharide transport-deficient *S. cerevisiae* strain EBY.VW4000 (Figure 4). On five different hexoses (maltose, glucose, galactose, fructose and mannose), *ChHxt1* to *ChHxt4*-expressing yeast cells could utilize maltose, fructose and mannose, but grew weakly on glucose. *ChHxt5*-expressing cells were only able to grow well on maltose and utilized fructose weakly. Only ChHxt6-expressing cells could grow on all five hexoses, demonstrating that *ChHxt6* is a central hexose transporter and crucial for hexose uptake.

### 2.6. Thr169 Position in ChHxt6 Is Vital for Hexose Transport and Virulence

**Figure 5:**
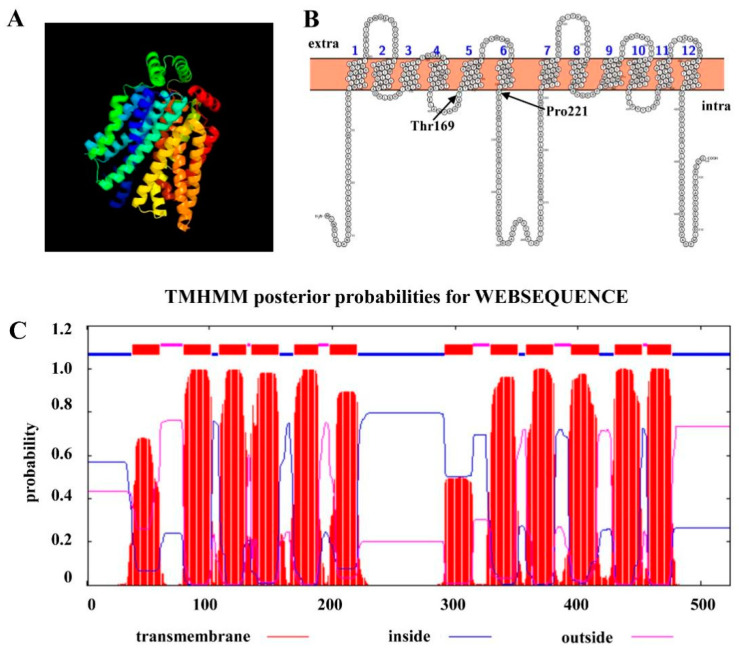
*ChHxt6* gene coding a hexose transporter protein. (**A**) 3D-model of ChHxt6 predicted by SWISS-MODEL. (**B**) Amino acid sequence and putative topology of ChHxt6p are shown in the membrane. (**C**) TMHMM posterior possibilities of amino acid in ChHxt6 by WEBSEQUENCE. The red lines represent the transmembrane part, the blue lines represent the intracellular part, and the purple lines represent the extracellular part.

After we identified ChHxt6 as a new central hexose transporter, we further analyzed the hexose transport structures of ChHxt6. The 3D structure of ChHxt6 protein was predicted with SWISS-MODEL, and the results revealed that the protein contains 12 transmembrane domains (TMD) (Figure 5A). There is a large intracellular loop between TMD6 and TMD7, which is typical for hexose transporters (Figure 5B,C). In the hxt1 protein of *U. maydis*, the Thr169 and Pro221 positions have been reported to be crucial for transport activity (Huser et al., 2015). To explore functions of hexose transport structure of ChHxt6, *ChHxt6(T169S)^m^*, *ChHxt6(P221L)^m^* and *ChHxt6(T169S, P221L)^m^* point mutants were generated by PCR-based site-directed mutagenesis. All ChHxt6 point mutants were verified by PCR with *ChHxt6*-KF/*ChHxt6*-KR primers (Table S1), following Sanger sequencing of the PCR products.

**Figure 6:**
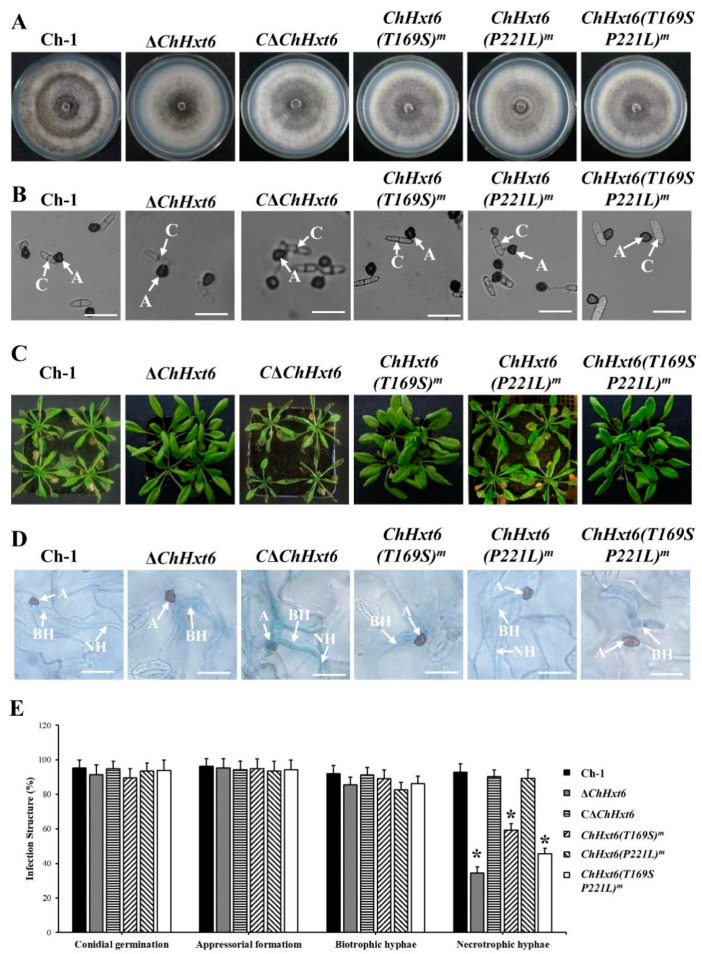
Virulence tests of *ChHxt6* site-directed mutants. (**A**) Colony morphology of the wild-type, *ChHxt6* knockout mutants, complementation strain and mutants from site-directed mutagenesis. All strains were cultured for 7 days on PDA plates in darkness at 25 °C. (**B**) Conidial germination of *ChHxt6* mutants derived from site-directed mutagenesis. Conidia of the strains were cultured on plastic coverslips for 24 h, and germination of conidia was observed under microscope. A: appressoria; C: conidia. Bar = 15 μm. (**C**) Virulence tests of *ChHxt6* mutants. (**D**) The infection structures generated at 48 h post incubation. The Arabidopsis leaves inoculated by conidia of wild type and mutants were collected at 48 h post inoculation. Infection structures were observed under microscope. Inoculated leaves were treated with methanol: chloroform: glacial acetic acid (6:3:1, *v*/*v*/*v*) and stained with trypan blue (0.4%). A: appressoria; BH: biotrohic hyphae; NH: necrotrophic hyphae. Bar = 20 μm. (**E**) Generation rate of infection structures. To calculate infection structure generation rate, at least 100 infection structure were observed, and each test was repeated three times. Bars show means and standard deviations of generation rates of infection structures, respectively. Asterisks represent significant differences between the Ch-1 and other strains by LSD test at *p* = 0.05.

In fungal morphology tests, there was no difference in colony morphology, vegetative growth or appressorial formation between the wild-type and all point mutants (Figure 6A,B). In *in planta* inoculation assays at 4 dpi, virulence of the point mutants *ChHxt6(T169S)^m^-5* and *ChHxt6(T169S, P221L)^m^-2* was decreased significantly compared to Ch-1 (Figure 6C). The virulence of *ChHxt6(P221L) ^m^-27* was similar to that of the wild-type (Figure 6C). Observation of infection structures suggested that formation rates of necrotrophic hyphae of *ChHxt6(T169S) ^m^-5 and ChHxt6(T169S, P221L) ^m^-2* were lower than that of the wild-type but higher than Δ*ChHxt6-2* (Figure 6D,E). Overall, these results suggest that Thr169 position in ChHxt6 is vital for virulence and necrotrophic hyphae formation.

**Figure 7:**
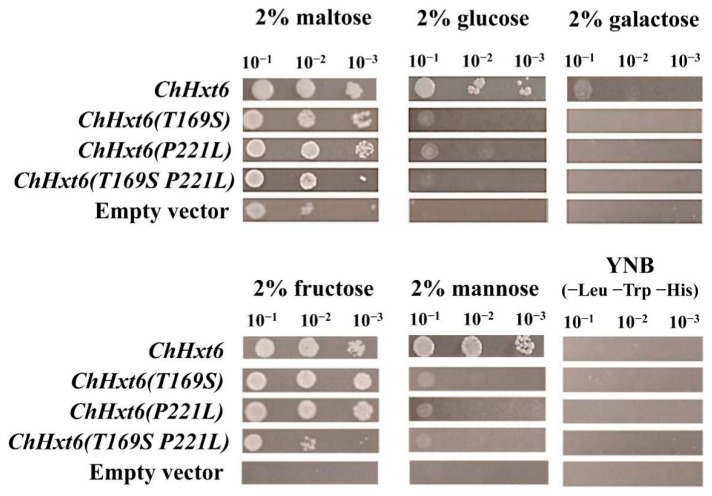
Growth of the *Saccharomyces cerevisiae* strains EBY.VW4000 and EBY.VW4000 expressing ChHxt6 site-mutation variants with amino acid exchanges on media containing different monosaccharides. Growth was observed after incubation at 28 °C for three days.

In hexose uptake assays, *ChHxt6(T169S)^m^*, *ChHxt6(P221L)^m^* and *ChHxt6(T169S, P221L)^m^* expressing yeast cells grew weaker than *ChHxt6*-expressing cells on all hexoses (Figure 7). These indicated that both Thr169 and Pro221 positions are necessary for hexose transport.

## 3. Discussion

Insertional mutagenesis is an effective method to identify novel genes involved in plant infection-related morphogenesis and pathogenicity in fungal pathogens, including *C. higginsianum* [2]. Here, we identified a new virulence gene, *ChHxt6* in *C. higginsianum*, using *Agrobacterium*-meditated random insertional mutagenesis. With BLAST against the *C. higginsianum* genome database, we found that there were 42 homologs of *ChHxt* in *C. higginsianum*. Since *CgHXT1* to *CgHXT5* in *C. graminicola* might have conserved functions during fungal pathogenesis [14], putative orthologs *ChHxt1* to *ChHxt5* from *C. higginsianum* seemed to be functionally different (Figure S2) and were chosen for biological function analysis. Among the six *ChHxt* genes, only *ChHxt4* and *ChHxt6* were required for fungal virulence. In published RNA-seq data [16], examination of the penetration, biotrophic and necrotrophic stages showed that *ChHxt4* and *ChHxt6* were highly expressed in necrotrophic stages, which was consistent with our RT-qPCR results. In the phylogenetic tree, *ChHxt6* was identified as a new hexose transporter whose homologs have not been explored in other fungi. *ChHxt4* is a putative ortholog of *MoST1* in *M. oryzae* and *CgHXT4* in *C. graminicola*, and CgHXT4 has been reported to be a sugar sensor [15]. MoST1 in *M. oryzae* is important for conidial production and hyphal melanization, but not for virulence. However, the *ChHxt4* deletion mutants in this study were defective in virulence, and these results suggest that the biological functions of Hxt orthologs are not conserved among different fungi.

In *S. cerevisiae*, growth defects were found after at least six hexose transporter genes were knocked out [6], or when either *Snf3p* or *Rgt2p* was knocked out, since these two sensors regulate the expression of some transporters [4,5]. In this study, deletion of single hexose transporters in *C. higginsianum* did not show obvious growth defects. These suggest that various hexose transporters in *C. higginsianum* might play overlapping and redundant functions. Virulence of *C. higginsianum* seemed to be sensitive to hexose uptake. Deletion of *ChHxt6* caused reduced virulence to *Arabidopsis*, which could be explained by defects in hexose uptake within the host plant. Similar situations have been proposed for the Hxt1-like transporters from *U. fabae* [8].

Expression of *ChHxt6(T169S)*, *ChHxt6(P221L)*, and *ChHxt6(T169S, P221L)* in Δ*ChHxt6* resulted in reduced virulence. In previous works, the mutation analogous to T169S was known to induce constitutive active glucose signaling in the hexose sensors of several yeasts [3,5], probably conferring a conformational stage that mimics the structure after binding of glucose. Thus, we assumed that the T169S position in ChHxt6 is important for transport activity, thereby affecting the development of infection structures.

Overall, functions of *ChHxt6* seemed to be unique among hexose transporter family genes in *C. higginsianum*. We demonstrated that *ChHxt6* is needed for hexose uptake and that hexose uptake might be involved in the necrotrophic stage of *C. higginsianum* during the infection process. This study demonstrated that ChHxt6 is a novel hexose transporter in hemibiotrophic fungi, and it affects the virulence of *C. higginsianum* through influencing the uptake of hexose in the necrotrophic stage.

## 4. Materials and Methods

### 4.1. Strains, Vectors and Plants

The *C. higginsianum* strain Ch-1, isolated from diseased tissues of *Brassica campestris*, was kindly provided by Prof. Yangdou Wei, University of Saskatchewan, Canada. Mycelia were placed on potato dextrose agar (PDA) plates and cultured in darkness for 7 days at 25 °C for conidial production. The *Escherichia coli* strain DH5α (TaKaRa, Dalian, China) was used for cloning. Vectors pMD18T-HYG and pNeoP3300III were used for gene disruption and complementation vector construction. The *Agrobacterium tumefaciens* strain EHA105 was used for transformation and stored at −80 °C in 20% glycerol (*v*/*v*) as a bacterial suspension [17].

The *Saccharomyces cerevisiae* mutant strain EBY.VW4000, which lacks all 20 identified hexose transporters was grown in a YNB medium (0.67% yeast nitrogen base supplied with tryptophan histidine and leucine) with 2% maltose at 29 °C [18]. Complementation studies in EBY.VW4000 were carried out on a YNB medium containing a set concentration of each hexose separately.

The *Arabidopsis thaliana* ecotype Col-0 which is susceptible to *C. higginsianum* was used in virulence assays. *Arabidopsis* seeds were sown on the surface of a peat-based compost and placed in a growth chamber at a 16 h/8 h (day/night) photoperiod with temperatures of 22 and 18 °C, respectively.

### 4.2. Molecular Identification of T-DNA Insertion Site from a Virulence-Deficient Mutant

After inoculation on *A. thaliana* Col-0, a virulence-reduced mutant G256 was screened from a *C. higginsianum* T-DNA insertion library. Flanking sequences of the insertion site were isolated with the inverse PCR strategy [17]. PCR products were cloned into the vector pMD18-T (TaKaRa, Dalian, China) and sequenced. The sequences were searched against the *C. higginsianum* genome database to obtain the full sequence of the target gene.

### 4.3. Deletion and Complementation of ChHxt Genes

Targeted deletion of *ChHxt* genes was achieved through homologous recombination. The total DNA of the *C. higginsianum* wild-type strain Ch-1 was isolated using a CTAB method [19]. The primers *ChHxt*_F1FP/*ChHxt*_F1RP and *ChHxt*_F2FP/*ChHxt*_F2RP (Table S1) were used to amplify about 1000 bp upstream (F1) and downstream (F2) flanking sequence of *ChHxt* genes, respectively. The fragments of *ChHxt*_F1 with *Hin*dIII/*Sal*I and *ChHxt*_F2 with *Xba*I/*Kpn*I were inserted into the corresponding restriction sites of the vector pMD18-HYG, resulting in the initial vector pMD18-F1-HYG-F2. After that, the F1-HYG-F2 cassette with *Hin*dIII/*Kpn*I was cloned into pneoP3300III, resulting in the disruption vector P3300neo-*ChHxt*.

The vector p3300neo-*ChHxt*, was transformed into *A. tumefaciens* EHA105 using electroporation, and then conidia of the wild-type were transformed with vector p3300neo-*ChHxt* by *A. tumefaciens*-mediated transformation (ATMT) [20]. To screen *ChHxt* knockout mutants, the transformants were grown on PDA supplied with 50 μg/mL of hygromycin (Merck KGaA, Darmstadt, Germany) and 500 μg/mL of cephalosporin (Amresco, Solon, OH, USA), and then sub-cultured on PDA supplemented with 150 μg/mL antibiotic G418 (Amresco, Solon, OH, USA) and 500 μg/mL of cephalosporin (Amresco, Solon, OH, USA). Transformants which grew on PDA plates supplemented with hygromycin but did not grow on PDA supplemented with G418 were selected as candidate knockout mutants. These were further verified by PCR amplification with *ChHxt*-KF/*ChHxt*-KR and HphSP/HphAP primers (Table S1).

To confirm that the phenotypes of the *ChHxt* knockout mutants were due to the targeted gene deletion, the knockout mutant Δ*ChHxt4-5* or Δ*ChHxt6-2* was complemented with a DNA fragment containing the complete coding region of *ChHxt4* or *ChHxt6* gene. The DNA fragment including the whole coding region of *ChHxt6* and 1.5kb upstream sequence, which is considered as promoter, were amplified with *ChHxt*-comFP/*ChHxt*-comRP primers (Table S1), and this fragment was cloned into the pneoP3300III vector with ClonExpress II One Step Cloning Kit (Vazyme, Nanjing, China). To obtain the *ChHxt6* complementation transformants, conidia of Δ*ChHxt* mutants were transformed with vector p3300neo-*ChHxt*-com by *Agrobacterium tumefaciens*-mediated transformation (ATMT). The complement transformants were screened on PDA containing 150 μg/mL G418, and further confirmed using PCR with gene-specific primers, *ChHxt*-KF/*ChHxt*-KR (Table S1).

### 4.4. Phenotype Analysis

Fungal mycelia from the edge of a 7-day-old colony were collected to observe hyphal tips by light microscopy. Conidial suspension droplets (10 μL) were also spotted onto microscope plastic coverslips in 9-cm-diameter petri dishes, lids replaced, and incubated at 25 °C. After 12 h, the conidial germination and appressorial formation were examined by light microscopy (Nikon, Tokyo, Japan).

### 4.5. Virulence Assay and Plant Infection Observation

The strains were propagated on PDA for 7 days at 25 °C. Sterile distilled water was added to each plate and gently scraped. The liquid was collected and conidia were harvested by passing through four layers of lens paper to remove debris and mycelium. In intact plant assays, conidial suspensions (1 × 10^6^ spores/mL) were sprayed onto 4 to 5-week-old *Arabidopsis* plants. The plants treated with sterile deionized water served as control. The inoculated plants were incubated at 25 °C and 100% RH in a plant growth chamber (18-h photoperiod and a flux rate of 40 μmol‧m^−2^‧s^−1^). Symptoms were recorded daily. To observe fungal infection process, inoculated leaf tissues were cleared in a solution of methanol: chloroform: glacial acetic acid (6:3:1, *v*/*v*/*v*) for 24 h and stained with trypan blue (0.4%) for 10 h. Inoculated leaves were collected at 24, 48 and 72 hpi for light microscopy observation and formation rate of infection structure were calculated.

### 4.6. Site-Directed Mutagenesis

Open reading frames of *ChHxt6 (T169S)*, *ChHxt6 (P221L)*, and *ChHxt6 (T169S, P221L)* were generated by PCR-based site-directed mutagenesis [21]. These sequences were first subcloned into the T-cloning vector pMD18-T (TaKaRa, Dalian, China), and then ligated with the vector pneoP3300III. The final vectors were transformed into the *C. higginsianum* by ATMT. After expression in *S. cerevisiae*, sequences of *ChHxt6*, *ChHxt6 (T169S)*, *ChHxt6 (P221L)*, and *ChHxt6 (T169S, P221L)* were amplified and cloned into plasmid pMD18-T. Primer sequences are presented in Table S1.

### 4.7. Expression of ChHxts in Yeast

For heterologous expression of *ChHxt* genes and *ChHxt6* site-directed mutant genes in *S. cerevisiae*, the coding region of *ChHxt* genes were amplified from the cDNA of Ch-1 and *ChHxt6* site-directed mutants using primers containing *Spe*I and *Cla*I restriction sites (ExpHxtF/ExpHxtR, Table S1) with the high-fidelity Platinum Taq DNA Polymerase (Invitrogen, Carlsbad, CA, USA). The amplification products were subcloned into the pMD18-T vector (TaKaRa, Dalian, China) by transformation into *Escherichia coli* DH5α cells (TaKaRa, Dalian, China). The sequences transformed into pDM18-T were verified by PCR using *ChHxt* specific primers and digested with *Spe*I and *Cla*I, and then the fragments were ligated into the yeast expression vector pEBY using T4 DNA Ligase (TaKaRa, Dalian, China). Plasmid DNA of pEBY-*ChHxts* was extracted using the Qiagen plasmid extraction kit (QIAGEN, Dusseldorf, Germany), and correct insertion of the fragments were verified by sequencing the cDNA/vector junctions (Tsingke, Beijing, China). The plasmids of pEBY-*ChHxts* were transformed into chemically competent cells of *S. cerevisiae* strain EBYVW.4000. The yeast transformants expressing the *ChHxt* genes were verified by PCR. Yeast transformants were selected for the ability to grow on selective YNB medium supplied with appropriate amino acids and 1% maltose [22]. Some of them were subsequently transferred to the same medium containing only one hexose as the sole carbon source. Growth of the yeast strain expressing *ChHxt* genes were compared to the strain harboring plasmid pEBY without insertion.

### 4.8. DNA/RNA Manipulation, Southern Blotting, RT-PCR and RT-qPCR

Genomic DNA was extracted using a CTAB method [18]. Southern blot analysis was conducted based on protocol of the Amersham Gene Images Alkphos Direct Labeling and Detection System (GE Healthcare, Amersham, UK). The probe DNA was amplified from the genomic DNA of the wild-type strain with labeled primers Probe-s/Probe-a (Table S1).

The *Arabidopsis* leaves inoculated with *C. higginsianum* at 24, 48 and 72 hpi were collected, flash frozen in liquid nitrogen, and stored at −80 °C. RNA isolation was carried out using the TRIzol^®^ Plus RNA Purification Kit (Invitrogen, Carlsbad, CA, USA), and potential DNA contamination was removed by DNase I treatment (RNase Free) (TaKaRa, Dalian, China) following the manufacturer’s instructions. First-strand cDNA was synthesized with the Revert Aid first strand cDNA Synthesis kit (Fermentas, St. Leon-Rot, Germany) following the manufacturer’s instructions. RT-PCR was used for confirmation of deletion and complementation mutants following the methods in previous work [23]. Expressions of *ChHxt* genes at different infection stages were analyzed by Real Time-PCR, and α-tubulin (*ChαTUB*, CH63R_12878) was used as the reference gene. The primers used in RT-qPCR assays are listed in Table S1. Each experiment was repeated three times.

### 4.9. Bioinformatics

The amino acid sequences of the *S. cerevisiae* Hxts were downloaded from NCBI (http://www.ncbi.nlm.nih.gov/, accessed on 1 May 2018). The Hxt homologues in *C. higginsianum* were located in the *C. higginsianum* genome using BLASTP (v2.2.31+) [24]. Open reading frames were further analyzed with FGENESH (Softberry Inc., Mount Kisco, NY, USA) [25]. The software SMART was used to predict conserved domains of proteins (http://smart.embl.de/, accessed on 5 April 2021) [26]. Multiple sequence alignment was conducted with Clustal X (version 2.0, http://www.clustal.org/clustal2/, accessed on 5 April 2021), and a phylogenetic tree was constructed using the Neighbor-Joining method with the MEGA software (version 7.0, http://www.megasoftware.net/, accessed on 5 April 2021) [27]. Transmembrane structures of putative Hxt proteins were predicted using CCTOP (version 2.0, http://cctop.enzim.ttk.mta.hu/?_=, accessed on 5 April 2021) [28].

### 4.10. Statistical Analysis

The statistical analysis software DPS (Date processing system, version 3.01, Beijing, China) was used to analyze all data from the quantitative assay with ANOVA (analysis of variance). When significant treatment effects were found (*p* < 0.05), the means were further separated using the test of least significant difference (LSD, *p* = 0.05).

## 5. Conclusions

In our study, six hexose transporter (Hxt) genes in phytopathogenic fungi *C. higginsianum* were identified and functionally analyzed. The six *ChHxt* genes were knocked out and the mutants were tested for colony morphology, conidial germination, and virulence to *Arabidopsis*. In addition, heterologous expression of *ChHxt1-6* and *ChHxt6* site-directed mutations in yeast were conducted for hexose uptake analyses. A total of 42 putative hexose transporters were identified in *C. higginsianum*, and *ChHxt1-5* was found as an ortholog of *CgHXT1-5* in *C. graminicola*. *ChHxt6* was identified as a novel hexose transporter in fungi. *ChHxt4* and *ChHxt6* are needed for full virulence of *C. higginsianum*, and the T169S and P221L amino acid sites are crucial for the function of ChHxt6.

## Data Availability

Not applicable.

## Supplementary Materials

The following are available online at https://www.mdpi.com/article/10.3390/ijms22115963/s1, Table S1: Primers used in this study. Figure S1: Gene knockout of *ChHxt* genes. Figure S2: Multi-alignment of ChHxt1p to ChHxt6p. The amino acid sequences of ChHxt1p to ChHxt6p were aligned with DNAMAN (v9). Homology parts were highlighted with dark shading.
