# Supplementary material for: A Novel Hexose Transporter ChHxt6 Is Required for Hexose Uptake and Virulence in Colletotrichum higginsianum
Source: Int J Mol Sci. 2021 May 31;22(11):5963. doi: 10.3390/ijms22115963 (PMC8199336; doi:10.3390/ijms22115963)
Supplement: Supplementary file 1 [file ijms-22-05963-s001.zip › ijms-1198172-supplementary.pdf]

Table S1 Primers used in this study

Primer name	Sequence (5'-3')	Remark
ChHxt1F1FP	5`-TGCATTTCCTCGTAAATTCTGGT-3`	Clone ChHxt1F1
ChHxt1F1RP	5`-CCAAGGAATGCATCTTCAAGCA-3`	
ChHxt1F2FP	5`-TGCACCACTCCTTGATGACC-3`	Clone ChHxt1F2
ChHxt1F2RP	5`-CCTCAAGTCAACACGGAGCA-3`	
ChHxt2F1FP	5`-AAACAGAGCCTCCACGATGC-3`	Clone ChHxt2F1
ChHxt2F1RP	5`-GGTTCCACACAAGGGCCTAA-3`	
ChHxt2F2FP	5`-TCTAACTGGTAAAGCTCCTCAGA-3`	Clone ChHxt2F2
ChHxt2F2RP	5`-ATCCACACCAAACATCCGCA-3`	
ChHxt3F1FP	5`-CTGGCCGTTAATCACGCTCT-3`	Clone ChHxt3F1
ChHxt3F1RP	5`-GAGACGCGGAAAGTCTCAG-3`	
ChHxt3F2FP	5`-ACCCATTTTCTCTTATGGGCCT-3`	Clone ChHxt3F2
ChHxt3F2RP	5`-CACGCCTTTTCTTCCCATGC-3`	
ChHxt4F1FP	5`-AGGCATCCAACACCACCTTATT-3`	Clone ChHxt4F1
ChHxt4F1RP	5`-GATCGTTCCTGCCTGGTTCA-3`	
ChHxt4F2FP	5`-CCTCAAGTCAACACGGAGCA-3`	Clone ChHxt4F2
ChHxt4F2RP	5`-AAACAGAGCCTCCACGATGC-3`	
ChHxt5F1FP	5`-GGTTCCACACAAGGGCCTAA-3`	Clone ChHxt5F1
ChHxt5F1RP	5`-TCTAACTGGTAAAGCTCCTCAGA-3`	
ChHxt5F2FP	5`-ATCCACACCAAACATCCGCA-3`	Clone ChHxt5F2
ChHxt5F2RP	5`-CTGGCCGTTAATCACGCTCT-3`	
ChHxt6F1FP	5`-CCTCAAGTCAACACGGAGCA-3`	Clone ChHxt6F1
ChHxt6F1RP	5`-AAACAGAGCCTCCACGATGC-3`	
ChHxt6F2FP	5`-GGTTCCACACAAGGGCCTAA-3`	Clone ChHxt6F2
ChHxt6F2RP	5`-TCTAACTGGTAAAGCTCCTCAGA-3`	
HPH-UP	5`-CACGCCTTTTCTTCCCATGC-3`	
HPH-DOWN	5`-ATCCACACCAAACATCCGCA-3`	
ChHxt1-KFP	5`-ATCCACACCAAACATCCGCA-3`	Validation of the ChHxt1 knockout mutants
ChHxt1-KRP	5`-CTGGCCGTTAATCACGCTCT-3`	
ChHxt1-UP	5`-TGCAGTGACAGATGAGCACAA-3`	
ChHxt1-DOWN	5`-ACTCTCCTGAGCTCCACAGT-3`	
ChHxt2-KFP	5`-CTGGCCGTTAATCACGCTCT-3`	Validation of the ChHxt2 knockout mutants
ChHxt2-KRP	5`-GAGACGCGGAAAGTCTCAG-3`	
ChHxt2-UP	5`-TGCACCACTCCTTGATGACC-3`	
ChHxt2-DOWN	5`-CCTCAAGTCAACACGGAGCA-3`	
ChHxt3-KFP	5`-ACCCATTTTCTCTTATGGGCCT-3`	Validation of the ChHxt3 knockout mutants
ChHxt3-KRP	5`-CACGCCTTTTCTTCCCATGC-3`	
ChHxt3-UP	5`-CTGGCCGTTAATCACGCTCT-3`	
ChHxt3-DOWN	5`-GAGACGCGGAAAGTCTCAG-3`	
ChHxt4-KFP	5`-AGGCATCCAACACCACCTTATT-3`	
ChHxt4-KRP	5`-GAGACGCGGAAAGTCTCAG-3`	

ChHxt4-UP	5`-CAAACATGGCTAAGCAGGGC-3`	Validation of the ChHxt4 knockout mutants
ChHxt4-DOWN	5`-GTCACAAGGCCTCTGCTTTTG-3`	
ChHxt5-KFP	5`-ACCCATTTTCTCTTATGGGCCT-3`	Validation of the ChHxt5 knockout mutants
ChHxt5-KRP	5`-CACGCCTTTTCTTCCCATGC-3`	
ChHxt5-UP	5`-GTCACAAGGCCTCTGCTTTTG-3`	
ChHxt5-DOWN	5`-AGTTACACAAAGCCGTGACAA-3`	
ChHxt6-KFP	5`-AGGCATCCAACACCACCTTATT-3`	Validation of the ChHxt6 knockout mutants
ChHxt6-KRP	5`-GATCGTTCCTGCCTGGTTCA-3`	
ChHxt6-UP	5`-CCTCAAGTCAACACGGAGCA-3`	
ChHxt6-DOWN	5`-AAACAGAGCCTCCACGATGC-3`	
ChHxt6C-FP	5`-GGTTCCACACAAGGGCCTAA-3`	Complementation of ChHxt6
ChHxt6C-RF	5`-GGTCTTCCACGATGTAACGAAGCA-3`	
ChHxt6T169S-FP	5`-CTGACTATTATGGTCCTACTATTTTCA-3`	Create mutant ChHxt6T169S
ChHxt6T169S-RP	5`-TGAAAATAGTAGGACCATAATAGTCACAG-3`	
ChHxt6P221L-FP	5`-TTTTTCTACTACGGTGCCGTTATTTTCAAGTCA-3`	Create mutant ChHxt6P221L
ChHxt6P221L-RP	5`-TGACTTGAAAATAACGGCACCGTAGTAGAAAAA-3`	
ChHxt1FP	5`-ACACTGACATTGATCGCAGC-3`	Clone ChHxt1
ChHxt1RP	5`-GACAAGCTATGCAATGCCACA-3`	
ChHxt2FP	5`-CAAACATGGCTAAGCAGGGC-3`	Clone ChHxt2
ChHxt2RP	5`-GTCACAAGGCCTCTGCTTTTG-3`	
ChHxt3FP	5`-AGTTACACAAAGCCGTGACAA-3`	Clone ChHxt3
ChHxt3RP	5`-CCATATTAAGCACTGGACGAGC-3`	
ChHxt4FP	5`-ACTGACATTGATCGCAGCGG-3`	Clone ChHxt4
ChHxt4RP	5`-GTCACAAGGCCTCTGCTTTTG-3`	
ChHxt5FP	5`-TGTAAACAAGGTTAGAGTTCAGGT-3`	Clone ChHxt5
ChHxt5RP	5`-CCAGGGGAGTAACCATTTTTCG-3`	
ChHxt6FP	5`-AAACGAGGCAGACAAACATGC-3`	Clone ChHxt6 and ChHxt6 point mutants
ChHxt6RP	5`-TCTTCCACGATGTAACGAAGCA-3`	

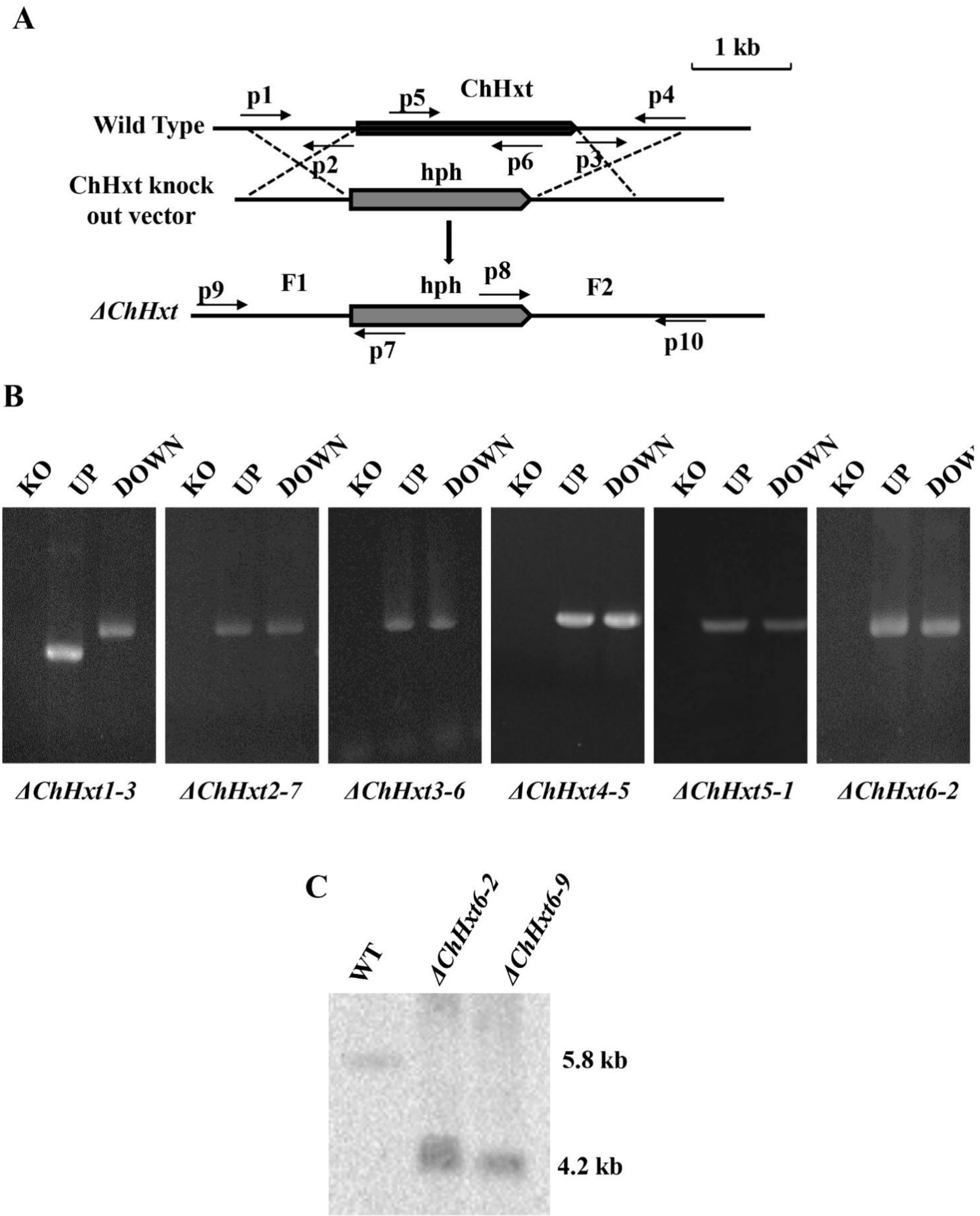

Figure S1 Gene knockout of ChHxts. A: Deletion strategy for ChHxt1~ChHxt6 genes in *C. higginsianum*. The p1 to p10 represents primers used for construction of ChHxt deletion vectors and validation of ChHxt mutants. p1: ChHxtF1FP, p2: ChHxtF1RP, p3: ChHxtF2FP, p4: ChHxtF2RP, p5: ChHxt-KFP, p6: ChHxt-KRP, p7: HPH-UP, p8: HPH-DOWN, p9: ChHxt-UP, p10: ChHxt-DOWN. B: Validation of ChHxt deletion mutants by PCR with primer pairs of p5/p6 (KO), p7/p9 (UP) and p8/p10 (DOWN). C: Validation of ChHxt6 knockout mutants by Southern blotting.

```
ChHxt1p  .......................MG..IKPAGVPGKAWPAIAIG.FFVAFGGLLFGYDTGTINGILEMSYWQRLFSTGYVNPAGNPDVSPSQESAIVSIL   74
ChHxt2p  .....................MGLGKLSVRINGAECGAEALMLG.AITSIGGFLFGYDTGQISGMLLFSDFKKRFATGSLGPDGLPQWVPTTQSLMVSLM   78
ChHxt3p  .......MEKETGVTREPVQGLAVTD.DVNAIEAPVTWKAYLIC.AFASFGGIFFGYDSGYINGVLGSSIFIEAVE.....GPGATSIGESDTSLIVSIL   86
ChHxt4p  ...................MAIAMGW.QKPDNVAGSSAPAIMVG.LFVASGGLLFGYDTGTINGILSMTAFRRDFTTGYT.ENGLPAISPAESSIIVAIL   78
ChHxt5p  MQFPKLGGKGVEHKDRTDADAPTDSNSVDLVAEQKVTFAACFLG.LVASIGGFMFGYVSGQISGFFLMEDFMERFGE...PQNGTYIFSAARQGTIVGLL   96
ChHxt6p  ...MGWNLKLKNSDDRHALESTTPSTTKTVSWLKDPGLRPLNFGLFFLLFGDVAHGFDGSLINNLQQINKWQEDFD..........HPRKSLLGALSASY   87

ChHxt1p  SAGTFFGALASPLLADSIGRRLGLAASCWVFNLGVVLQTIA...TDIPVFLAGRFFAGFGVGLISALVPLYQSETAPKWIRGAIVGAYQWAITIGLLLAA  171
ChHxt2p  SIGTLIGALSGAYTADWWGRRRSLSFGVAIFIIGNIIQISA..MNSWVHMMVGRFIAGLGVGNLSIGVPMFQSECSPREIRGAVVASYQLLITFGILISN  176
ChHxt3p  SCGTFFGALIAGDLADMMGRKWTVILGCLIYMIGVIIQMITNVNTALGPIVAGRLIAGIGVGFESAVVILYMSEISPKKVRGALVSGYQFCITIGLLLAA  186
ChHxt4p  SAGTILGALLAAPIGDSWGRRISLILSVGVFSFGGIFQVCA...HDIPMLLVGRFFAGVGVGSISVLVPVYQSEMAPKWIRGTLVCAYQLSITIGLLSAS  175
ChHxt5p  CIGCLVGALTAGKLADTLGRRLTISLFAFFSCIGIVIEISS..TNKWYQFAIGRLVNGVGIGALSVVVPMYQSESTPAIIRGVIVSTYQLFITLGIWLAE  194
ChHxt6p  WIGNILGVVLIPLVADRLGRRIAIAAGSLLCIGGAAICAAT...TSNGAFIAGRILLGMGGVMCSSVSTVLMTELAYPAHRATATALSSTTYSVGAILAA  184

ChHxt1p  IVNNATHNRQDT.GSYRIPIAIQFAWSIILFVGMIILPETPRFLIKSGHVDRATKALAKLRRL..PENDPYVAEEIAEIKANHDFETSIGT.....ASYL  263
ChHxt2p  LINFGVRNFQDSDASWRIVIGLGIGFSLPLGLGILFVPESPRWLAGRQDWEGSRMALARLRGLKDDPHCDLVEKDLQEMFKVIEEESKTGY.....GTWA  271
ChHxt3p  CVVYATKDRTDT.GSYRIPIAIQFPWAIILGGGLMLFPDSPRYFVKKGRLADAARSLSRLRGQ..PENSEYIQVELAEIVANEEYERQLVPNTTWFGTWA  283
ChHxt4p  FVNILTEKLQTA.AAYRVPLGLQLVWAVVLAIGLLVLPETPRFLVKQGKPEAAGLSLSRLRRL..DITHPALLEELQEIIANHEYELTLGP.....DSYK  267
ChHxt5p  IVNFATHNKVGS.ASWRIPNGLGFAWALILGAGILLLPESPRYAFRQGREDEARRNIAKLAGV..EPNAMSVNAQIDDIRAKVAEESAGAD.....TSIW  286
ChHxt6p  WVAFGSFRIEDS.WAWRTPTLIQAFPSAMVLCGLFFLPESPRWLCSRGKEQTALDILAKYHGAG.NVDDAVVQHEYAEIRDTIEAEITQNKNP...FHLK  279

ChHxt1p  DCFR......PPVLKRQFTGMALQALQQLTGINFIFYYGTQYFQNS.GFSNGFVIGMITS...SINVVSTIPGMYAVDRWGRRPMLLWGAVGMCVSQFLV  353
ChHxt2p  ECFTGSS.GIPKTVYRTLLGISLHFIQQWTGVNFFFYYGATIFESA.GIEDPIMMQLILG...AVNVFCTFFGLYAVEKYGRRWPLFIGAVWQTAWLTVF  366
ChHxt3p  NCFKGSVFKANSNLRKTILGTSLQMMQQWTGVNFIFYYSTPFLQSTGAIDNTFLISLVFT...LVNVCSTPLSFWTVERFGRRTILLWGATGMLICQFLV  380
ChHxt4p  EIFYG....SPHLGRRTLTGCCLQMLQQLTGINFIMYYSTSFFDGA.KVENPYLKALIIN...IINVVSTIPGLLVIESWGRRKLLMVGAMGMAVCQLLI  359
ChHxt5p  EIFTG.....PRMLYRTILGVVLQAGQQLTGANFFFYFGTTVFSAT.GLSDSYVTQLILG...SVNVACTFGGLVVVKKSGRRMALIIGALWMMMCFFVY  377
ChHxt6p  ALFAT.....PGNRWRSFIIIWCGICKQWSGNGLVSYYLGSMLKSA.GITKQIETTLITATSQMFSFACSFAFAFLPARVGRRPLMLTSMAGMWIVFAVI  373

ChHxt1p  AMLG......TLTTGQDDAGKIIVYNLPAQKAAIAFVCIYIFFFASTWGPLAWVVNGEIFGLKTRAKSLSLSTATNWLLNWAIAYATPYLVNYGDGYANL  447
ChHxt2p  AAVG......VAMP........PETNSTTGIVMIVSACMFIASFASTWGPMCWCVIGETFPLRTRAKQASLATAGNWLGNFLIAFLTPYATA......GI  446
ChHxt3p  AIIGVTVGFNHTHPDPADATKSIANNIPAVNAQIAFIAIFIFWFASTWGPGAWVLIGEIFPLPMRSRGVALSTASNWLWNTIIAVITPYMVGENRG..NL  478
ChHxt4p  ASF.......TTAAGEN.......LQQAAQTILIVFCAVNIFFFAASWGPVCWVITSEIYPLKVRAKANSISTASNWLLNFGIAYGTPFMVGQGTGSADI  445
ChHxt5p  AFVGH.....FALDQND.....PQNTPTAGTVLVAFSCLFIAGFATTWGPLVWAVVAELYPARYRAPAMALATASNWLWNFLMSFFTRYITD......AI  461
ChHxt6p  TATS......GAYVETG........NRHASYTTVAFIYLYSGVHNLGWTGAQMLYIVEILPYTIRAKGMAMFSLVAGICGAFNTYVNPLGIA......AM  453

ChHxt1p  QSKIFFVWFACCFLCIAFVYFFIYETKGLTLEEVEELYAEVSVASKS.ANWRPATTFRERQAAEGDKAVPHNSDGDTAHHEKTVDA..............  532
ChHxt2p  GYSYGFVFAACNLAGAVVVWFFLYETKMLSLENVDRMYSDPSVKPYSSSKWVPPGYITRKQKDDSAFQRASVHSENTAVGGDQHKEKTSDDERPFSEHHT  546
ChHxt3p  KSSVFFVWGGLCSCALVYTYFLVPETKGLSLEQVDKMMEET.TPRTS.AKWVPHETFASQVARNG..VLDKVTVDNVERRSSNV................  558
ChHxt4p  GPKIFFLWGAFCILAVLFVWCMVFETSKISLEQIDEMYERVDYAWNS.TRFEPSWSFQQILDEGWSPSAQPPLEHELQQTQTNSTTQTTQTSHTNSNSAT  544
ChHxt5p  DYLYGLVFAGCCAALAVIVFFFVIESKDRTLEEIDTMYVQR.VNPITSAKWR..GEVRKNHHNESSSEAEKAEATV........................  534
ChHxt6p  SWKFYFFYVGWIIVQFVVVYLVFPETKGPSLEQIALLFDGK.DAQVGRVNAVADEMLDEKEGGVAVETRERQ............................  524

ChHxt1p  ...........................  532
ChHxt2p  KQEPLANNV..................  555
ChHxt3p  ...........................  558
ChHxt4p  STNTGATGNSTSAEAKMLSQMGNVDFS  571
ChHxt5p  ...........................  534
ChHxt6p  ...........................  524
```

Figure S2 Multi-alignment of ChHxt1p to ChHxt6p. The amino acid sequences of ChHxt1p to ChHxt6p were aligned with DNAMAN (v9). Homology parts were highlighted with dark shading.
